# A Treatment for Trench Fever

**Published:** 1919-01

**Authors:** 


*A Treatment for Trench Fever.* By Major J. E. SWEET and Lieutenant H. B. WILMER.

The authors state that their results are presented with a full knowledge of the fact that there is absolutely no treatment for trench fever.

A trial of collargol seemed worth while for the drug differs considerably from other colloid silvers, and there is no great evidence of particular worry about trench fever in the enemy literature. The authors present their results even though the number of cases is relatively small — the total is 35 cases — because of the uniformity of the results obtained; 100 o/o of 35 cases is somewhat better than 35 o/o of 100 cases. There has been no question in the minds of the clinicians, who have followed these cases, that the collargol was immediately followed by marked improvement and recovery. It has only been difficult to decide in some cases whether the collargol was merely an incident in the cause of the spontaneous recovery from the disease. Some cases do so recover, though by no [means the 90 o/o of the cases reaching the base reported by the British Trench Fever Committee. The authors have learned of no method of distinguishing a case which will spontaneously recover and one which will spend the next six months in hospital.

It will be noted that a pronounced reaction follows the intravenous injection of 10 cc. of a 1 o/o solution of collargol, made in sterile distilled water, and it is of interest that the patients often compare this reaction to their initial attack.

The authors have not always carried out the injection at two or three days' intervals. In some they have done this, and the reaction seems to be generally much less marked when the second injection follows the first after a short interval. They purposely did not carry out the two or three day injections in many cases, for it seemed a way to control the question of whether they were dealing in coincidents.

The authors have chosen the following seven cases to illustrate certain points.

1. Capt. A. M. C., U. S. A. Attack began Feb. 6th, 1918. Course marked by fever, recurring every seventh day, preceded by chills and accompanied by backache, headache and shin pain. Lost about 20 lbs. in weight. June 21, 10 cc. collargol. Has had no return of symptoms since, and has regained the lost weight. Has been under observation for 15 weeks since the collargol.

2. Pvt. S. Entered hospital bringing chart for Aug. 12-19. Aug. 26, collargol. Temperature fell to subnormal the next day, rose to normal Aug. 28, and remained normal until discharge, Sept. 11.

3. Pvt. H. Admitted 8/5/18. Patient said he had had chills and sweats, pain in head, back, and shins for five weeks, and the same sort of attack 14 months ago. Collargol 8/9/18. Observed until 8/22/18, with no return of fever or typical pain. Discharged complaining of weakness and stiffness, and pain in knee and wrist joints.

4. Pvt. H. Admitted to C. C. S. 8/13/18, complaining of headache, backache, pain in shins, and chills and fever. Admitted to hospital 8/22/18. 8/24/18, collargol. 8/28/18, out of bed, no discomfort. Observed to 9/4/18, no complaints except feeling weak.

5. Pvt. S. Attack began Aug. 4. Fever, chills, pain in head, back and shins, perspires easily. Fever evidently of the six-day ntermittent type. Collargol 8/14/18, following fever spike. Second injection 8/17/18 with no reaction. Observed to 8/31/18, no return of fever.

6. Pvt. E. Admitted to C. C. S. 8/19/18, to hospital 8/22/18. Collargol 8/24/18. Return of fever and pain 6 days later; 2nd injection collargol. Discharged 9/24/18.

7. Bugler J. Admitted 8/12/18, with usual symptoms and extreme tenderness of shins. Says he had similar attack in July, 1916; was 3 months in hospital and convalescent camp. In July, 1917, 2nd attack; spent 31 weeks in hospital, convalescent and training camps. Says present attack is more severe than either previous attacks. 8/15/18, collargol. 6 days later fever and pain recur; 2nd collargol 8/25/18. 8/28/18, out of bed, no aches or pain. Discharged 9/17/18, with no aches or pains and gaining weight.

